# Stability indicating green micellar liquid chromatographic method for simultaneous analysis of Metformin and dapagliflozin in their tablets

**DOI:** 10.1186/s13065-025-01537-8

**Published:** 2025-06-21

**Authors:** Aya A. Marie, Mohamed G. Yassin, Aya Roshdy

**Affiliations:** 1 Faculty of Pharmacy, Pharmaceutical Chemistry Department, Horus University, New Damietta, 34517 Egypt; 2 Zeta Pharma for Pharmaceutical Industries, Sadat City, Egypt; 3 Methodology Section Head, Zeta Pharma for Pharmaceutical Industries, Sadat City, Egypt; 4 Faculty of Pharmacy, Pharmaceutical Chemistry Department, Aya Roshdy Lecturer, Horus University, New Damietta, 34517 Egypt

**Keywords:** Micellar liquid chromatography, Stability indicating, Dapagliflozin, Metformin, Tablet dosage form, Greenness assessment

## Abstract

A green micellar liquid chromatography (MLC) technique was established for simultaneous determination of dapagliflozin (DAP) and Metformin HCl (MET) in their pure and tablet dosage forms. Stability studies were carried out for both drugs using different five degradation conditions. The chromatographic separation was achieved using BDS Thermo-Hypersil C8 (150 mm x 4.6 mm, 5 μm) column and hybrid micellar mobile phase composed of 50 gm of sodium lauryl sulphate in 500 mL purified water with 100mL of 2-Propanol and 3 mL triethylamine and then completed with purified water to 1000mL, and the mobile phase pH was 3.3. The (PDA) detector was used at 223 nm, 40 °C column temperature, 1mL/min flow rate and 20µL injection volume. The retention times of DAP and MET were (5.37 ± 0.4 min) and (7.64 ± 0.2 min), respectively. Linearity was constructed in ranges of (0.2–7 µg/mL) for DAP and (50–700 µg/mL) for MET. Mean percentage recoveries of DAP and MET were 99.88%±0.53 and 100.034%±0.60, respectively indicates good accuracy. The developed approach was validated according to ICH guidelines and efficiently applied for the analysis of DAP and MET in Dexigloflozin plus^®^ 5/500 tablets. For the greenness assessment the analytical eco-scale, Complex MoGAPI and AGREE methods were applied.

**Supplementary Information:**

The online version contains supplementary material available at 10.1186/s13065-025-01537-8.

## Introduction

In Egypt, diabetes mellitus is regarded as one of the most prevalent and rapidly spreading illnesses [1]. Most of anti-diabetic drugs are administered orally and act by regulating the levels of blood glucose [2]. The management of diabetes mellites frequently requires combination therapy, so the use of fixed-dose combination therapy is more preferred than single drug.

Dapagliflozin (DAP) (Figure S1 a) is a sodium-glucose co-transporter 2 (SGLT2) blocker. It utilized mainly for the management of diabetes mellites by blocking the glucose reabsorption in kidney and increase the glucose excretion in urine, DAP drops plasma glucose in an insulin-independent way [3]. The reported methods for analysis of DAP involving spectrophotometric [4–7], spectrofluorimetric [8, 9], HPLC [7, 10–12] and HPTLC [13–15] methods.

Metformin (MET) (Figure S1 b) is 1,1-Dimethylbiguanide N, N-dimethylimidodicarbonimidic diamide belongs to biguanide class. It’s the drug of choice for the type-2 diabetes mellites treatment [16]. It reduces the production of glucose in liver and suppresses triglyceride and cholesterol levels [16]. MET was analyzed using different approaches including spectrophotometry [17, 18], HPLC [19–22] and HPTLC [23, 24] techniques.

Dexigloflozin plus^®^ (5/500) tablet which is composed of 5 mg DAP and 500 mg MET manufactured by Zeta Pharma for Pharmaceutical Industries for treatment of diabetes mellitus. Numerous techniques were published for the simultaneous estimation of DAP and MET in their dosage forms including spectrophotometric [25–29], RP-HPLC [30–36], LC-MS [37–39] and HPTLC [31, 40] methods. By reviewing all reported RP-HPLC methods [34–40], it has been proven that there are only two reported stability indicating methods have been published for simultaneous analysis of DAP and MET [33, 35]. However, no micellar liquid chromatography (MLC) approach has been published or addressed in literature for the simultaneous quantitation of DAP and MET. The proposed (MLC) technique processes many advantages over these two methods; as the proposed method was used greener mobile phase. While the reported method [33] was used 40% acetonitrile and reported method [35] was used 75% methanol in their mobile phases. Also proposed method have wider linearity ranges for both drugs compared to reported method [33]. Micellar liquid chromatography (MLC) is an effective substitute to RP-HPLC with hydro-organic mobile phases. MLC is considered as “green” technique as it uses about 90% or more water in mobile phases. The MLC not producing hazardous wastes and have a low toxicity [41]. Most MLC use hybrid micellar mobile phases composed of a small amount of organic modifier with surfactant more than the critical micellar concentration (CMC) to increase the elution strength and efficiency [41].

The aim of the present work is to establish a first green stability indicating MLC technique for simultaneous analysis of DAP and MET based on hydro-organic mobile phase to enhance the efficiency and elution strength. Good agreement was established when the assay results of the developed MLC method were statistically compared to those of published approach [34]. Analytical eco-scale [42], Complex MoGAPI [43] and AGREE [44] methods were applied for assessment of the greenness of the proposed method.

## Experimental

### Apparatus and software

Shimadzu prominence-i^®^ series LC-2030 C 3D plus system (Shimadzu, Kyoto, Japan), equipped with RS thermostated column compartment, RS auto-sampler injector and quaternary RS pump. Lab solutions software^®^ (Shimadzu, Japan) was used for data acquisition. BDS Thermo-Hypersil C8 (150 mm x 4.6 mm, 5 μm) column. Jenway 3510 pH-meter (UK), Hettich centrifuge (Tuttlingen, Germany) and Rocker 811 lab vacuum pump (Lingya Dist., Kaohsiung city 802, Taiwan) were used. The nylon membrane filter 0.22 μm (Millipore, Ireland) was utilized for filtration of mobile phase.

### Chromatographic conditions

The estimation of both drugs was achieved on BDS Thermo-Hypersil C8 (150 mm x 4.6 mm, 5 μm) column and hydro-organic mobile phase composed of 50gm of sodium lauryl sulphate in 500mL purified water with 100mL of 2-Propanol and 3 mL triethylamine, completed to 1000mL with purified water). Orthophosphoric acid was used to adjust pH to 3.3. The flow rate was 1mL/min, 20µL injection volume and 40 °C column temperature were used. (PDA) detector was set at 223 nm which selected based on the ratio of each analyzed drug in the dosage form and the UV signal intensity of each analyzed drugs.

### Materials and reagents

DAP (99.95%purity) and MET (99.60% purity) were provided friendly from Zeta Pharma for Pharmaceutical Industries, El Menofia, Egypt. Dexigloflozin plus^®^ (5/500) tablets manufactured by Zeta Pharma for Pharmaceutical Industries and used for treatment of diabetes type 2. It’s composed of 5 mg DAP and 500 mg MET.

Methanol (HPLC grade) was bought from (Fisher, UK). Sodium lauryl sulphate (Inter. Trade Co, Japan). Analytical grade Orthophosphoric acid, 2-propanol and triethyl amine (Sigma-Aldrich).

### Stock and working standard solutions

2000 µg/mL MET and 1000 µg/mL DAP stock solutions were prepared separately by transferring accurately weighed 100 mg of MET in 50mL volumetric flask and 30.75 mg of DAP propanediol monohydrate (equivalent to 25 mg of DAP base) in 25mL volumetric flask. Both were dissolved using (50:50, water: methanol) solvent and diluted to the mark using the same solvent.

Working solutions of both drugs were prepared by serial dilution of stock solutions with the same solvent to attain 10 µg/mL DAP and 1000 µg/mL MET. All solutions were kept at 4 °C.

### Construction of calibration curves

Solutions within concentration ranges (0.2–7 µg/mL) of DAP and (50–700 µg/mL) of MET were prepared by transferring different volumes from DAP & MET working solutions to separate 10mL volumetric flasks. Solutions were diluted to the mark using mobile phase. 20µL were injected from each solution into chromatograph using stated separation parameters. Calibration curves were found by constructing the average peak area against different DAP and MET concentrations and the regression equations were computed.

### Procedure for analysis of Dexigloflozin plus^®^ (5/500) tablets

Twenty tablets of Dexigloflozin plus^®^ (5/500) were weighed and grained in mortar. Then into 100mL volumetric flask, a weight of the powdered tablets equivalent to the average weight of two tablets was transferred and 75mL solvent were added, swirl with sonication and shaking for (20 min). Then solution was cooled and diluted to the mark using solvent. The solution was centrifuged at (5000 rpm) for 15 min and (5mL) of supernatant was transferred to 100mL volumetric flask. The solution was diluted to the volume using solvent to attain 5 µg/mL DAP and 500 µg/mL MET.

### Solutions for forced degradation studies

Specificity of the developed approach was confirmed by stability studies which were assisted by comparing chromatograms non-stressed 5 µg/mL DAP and 500 µg/mL MET with degradation solutions which were exposed to five forced conditions. Solutions of 50 µg/mL DAP and 5000 µg/mL MET were prepared as working standard for degradation studies. Comparison was performed between non-stressed standard solutions and the response of each degradation solution to measure the decrease in the DAP or MET peak area and by the presence of new degradation peaks.

#### Alkaline degradation

For investigation of alkaline degradation; into two separate 50mL volumetric flasks, 5mL from each 50 µg/mL DAP and 5000 µg/mL MET solutions were transferred. Each volumetric flask contains 5mL of 0.1 N NaOH for (5 h.) in dark at 80 °C. The solutions were neutralized separately after mentioned time by adding 5mL of 0.1 N HCl and diluted to the marks using solvent to obtain 5 µg/mL DAP and 500 µg/mL MET.

#### Acid degradation

For investigation of acidic degradation; 5mL from each 50 µg/mL DAP and 5000 µg/mL MET solutions were transferred into two separate 50mL volumetric flasks, each volumetric flask contains 5mL of 0.1 N HCl for (5 h.) in dark at 80 °C. The resulted solutions were neutralized after mentioned time by adding 5mL of 0.1 N NaOH and diluted to the volumes using solvent to obtain 5 µg/mL DAP and 500 µg/mL MET.

#### Oxidation degradation

For oxidation degradation investigation, 5mL from each 50 µg/mL DAP and 5000 µg/mL MET solutions were transferred into two separate 50mL volumetric flasks, then these solutions were exposed to 5mL of 3% v/v H_2_O_2_ and kept at room temperature in dark for 5 h. Then solutions were diluted to the volumes using solvent.

#### Photo degradation

Photodegradation was investigated by transferring 5mL from each 50 µg/mL DAP and 5000 µg/mL MET solutions into two separate 50mL volumetric flasks. The solvent was used for dilution of these solutions up to the volumes. These solutions exposed to the day light for 6 h. After the specified time the solutions were injected into chromatograph.

#### Heat degradation

For heat degradation investigation, 5mL from each 50 µg/mL DAP and 5000 µg/mL MET solutions were transferred into two separate 50mL volumetric flasks and diluted to the marks with solvent then exposed to heat at 80 °C for 6 h.

## Results and discussion

**Fig. 1:**
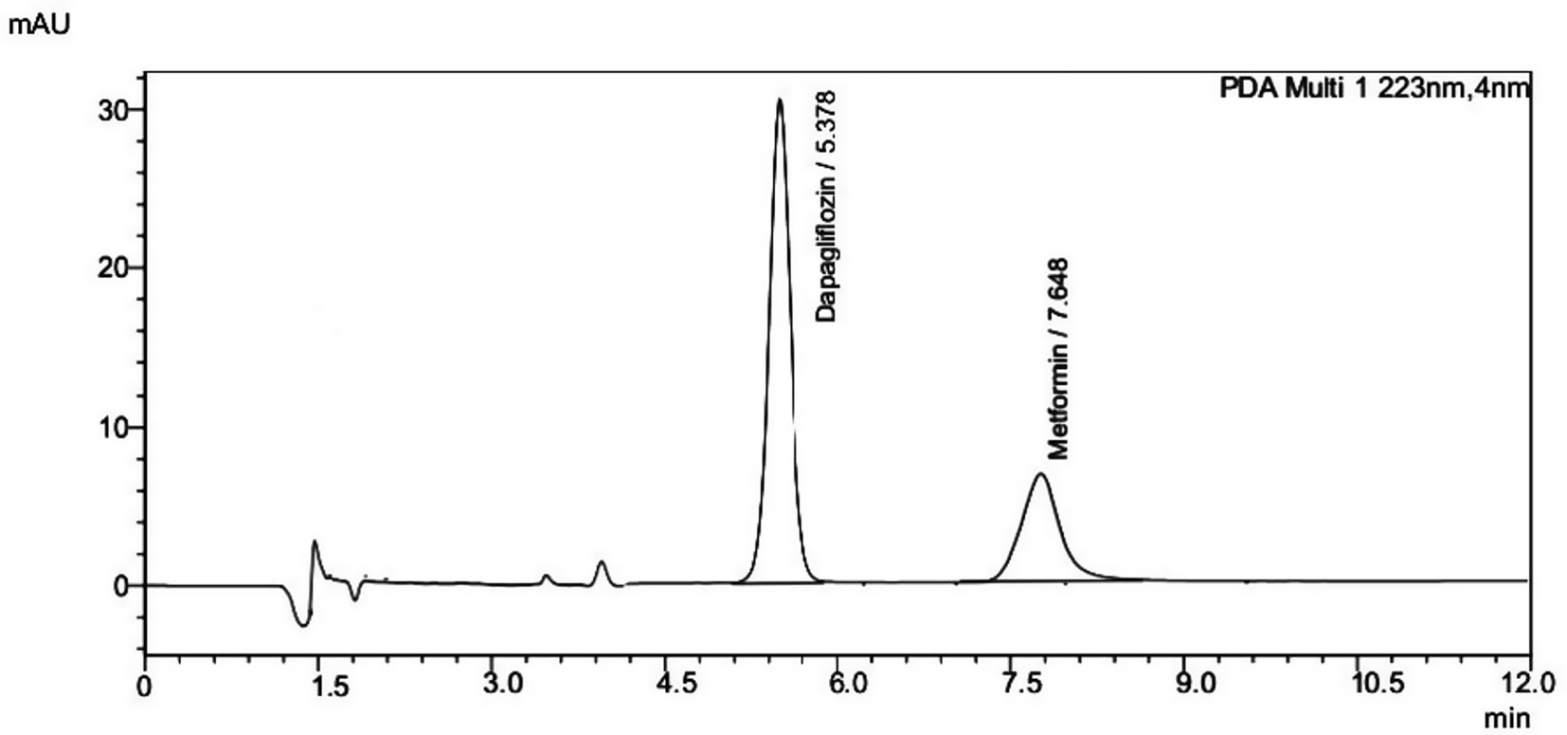
Typical chromatogram of (10 µg/mL DAP and 5 µg/mL MET) mixture in their pure forms under the specified separation conditions

The established MLC technique was applied for separation of DAP and MET in bulk and tablet forms in less than 10 min. The MLC was used to enhance the retention of MET peak by using SLS more than CMC to increase the efficiency and elution strength [41]. Figure 1 shows the retention times of DAP and MET which are (5.37 ± 0.4 min) and (7.64 ± 0.2 min), respectively.

MET is a highly soluble oral antidiabetic drug of small size and high cationic charge (at low PH) [45]. In all the previously reported methods, the MET was highly un-retained due to the highly polar nature. So, it’s expected to be eluted before DAP.

Using anionic ion pairing agent such as SLS is an approach for modulation of hydrophilicity of water‑soluble cationic drugs as MET at low pH via complexation with oppositely charged molecules [45]. In the proposed MLC technique, use of SLS at a concentration exceeding its CMC produces micelles that trap the MET-SLS hydrophobic non-polar complex into the micelles and make it more retained and eluted after DAP by selective competition between the micellar pseudo-stationary phase and the column stationary phase, giving selective separation between the two highly polar APIs (MET and DAP), while using a low %organic (20% Isopropyl Alcohol as an organic eluent).

### Method optimization

Parameters such as: mobile phase flow rate, column temperature, detection wavelength and mobile phase pH are known to affect the peak shape, retention time, capacity factor and number of theoretical plates. These parameters were investigated and evaluated to select the optimum separation conditions. The detection wavelength was selected based on dosage form ratio of each drug and the UV signal intensity of each drug. The wavelength at 223 nm is the wavelength that achieved the highest signal intensity for DAP which presents in low concentration in dosage form (ratio 5: 500, DAP: MET; respectively) and relatively lower MET signal intensity.

The values of column temperature were examined in range (25–40 °C) at constant pH 3.3 and 1mL/min flow rate. When column temperature was raised up to 40 °C, provided reasonable retention times and better peaks shape. So, 40 °C was efficiently selected as best column temperature. Different flow rate values were examined in range (0.9–1.5) mL/min. As the flow rate increase more than 1mL/min provided minimum resolution. While, flow rate less than 1mL/min causes too late retention times and peak broadening. So, 1mL/min was efficiently selected as the optimum flow rate. Different pH values were investigated between (3–6) at flow rate 1mL/min and column temperature 40 °C. The pH value 3.3 was selected efficiently for better separation and higher resolution. Table S1 presents the system suitability parameters for DAP and MET at the selected separation conditions.

### Method validation

The established approach was validated for determination of DAP and MET according to ICH guidelines [46].

#### Linearity and range

Linearity ranges were established for DAP and MET in concentration ranges (0.2–7 µg/mL) and (50–700 µg/mL), respectively. The regression parameters for DAP and MET were presented in Table 1 which confirm the good linearity for DAP and MET where r values more than 0.999.

#### Detection and quantitation limits

Using the following Eqs. (1) and (2), the LOD and LOQ values for DAP and MET were calculated regarding to the ICH guideline [46]. The results were presented in Table 1.

1 $$\:LOD\hspace{0.17em}=\hspace{0.17em}3.3\times\:\:S_a\:/b$$

2 $$\:LOQ\hspace{0.17em}=\hspace{0.17em}10\times\:\:S_a/b$$

where S_a_ is the standard deviation of y-intercept and b is slope of regression lines.

**Table 1:** Results of regression parameters for analysis of DAP and MET

Drug	DAP	MET
Concentration range (µg/mL)	0.2-7	50–700
r	0.9999	0.9993
a	1181.671	-782132.873
b	54609.272	75959.209
Sa	1074.143	378275.463
Sb	279.349	983.073
S(y/x)	1889.944	652513.343
LOD (µg/mL)	0.065	16.434
LOQ (µg/mL)	0.197	49.800

a, intercept; b, slope; r, correlation coefficient

S_a_, standard deviation of intercept; S_b_, standard deviation of slope;

S_y/x_, residual standard deviation

LOD, limit of detection; LOQ, limit of quantitation

#### Accuracy

Accuracy of the established approach was assessed by calculating of the mean percentage recoveries of DAP and MET of three determinations at three concentration levels within the linearity ranges. Concentrations (2.5, 5 and 6 µg/mL) for DAP and (250, 500 and 600 µg/mL) for MET. Table.S2 presents the resulted mean % recoveries for DAP and MET, all results are within compendial tolerance (98–102%).

#### Precision

Precision was confirmed by calculation of (S.D.) and (%RSD) of three injections of three concentrations for of DAP and MET on the same day for intraday precision and on three consecutive days for interday precision. Table S2 presents the S.D. and %RSD results, all values were less than 2 which confirm the good intra and interday precisions of the proposed approach.

#### Selectivity

The selectivity is confirmed by the comparison of the chromatograms of standard solution of DAP and MET in pure forms, test solution of Dexigloflozin Plus^®^ 5/500 mg tablet, solution of the inactive ingredients of Dexigloflozin Plus^®^ (Placebo solution) and the diluent used. As presented in Figure S2 there is no interference from the excipients with the determination of both drugs.

#### Robustness

The robustness was indicated by that the proposed method was remain unaffected by the small but deliberate variations of different parameters. Table S3 presents the calculated (S.D) and (%RSD) values of %recoveries and all results were less than 2.

### Results of forced degradation studies

**Fig. 2:**
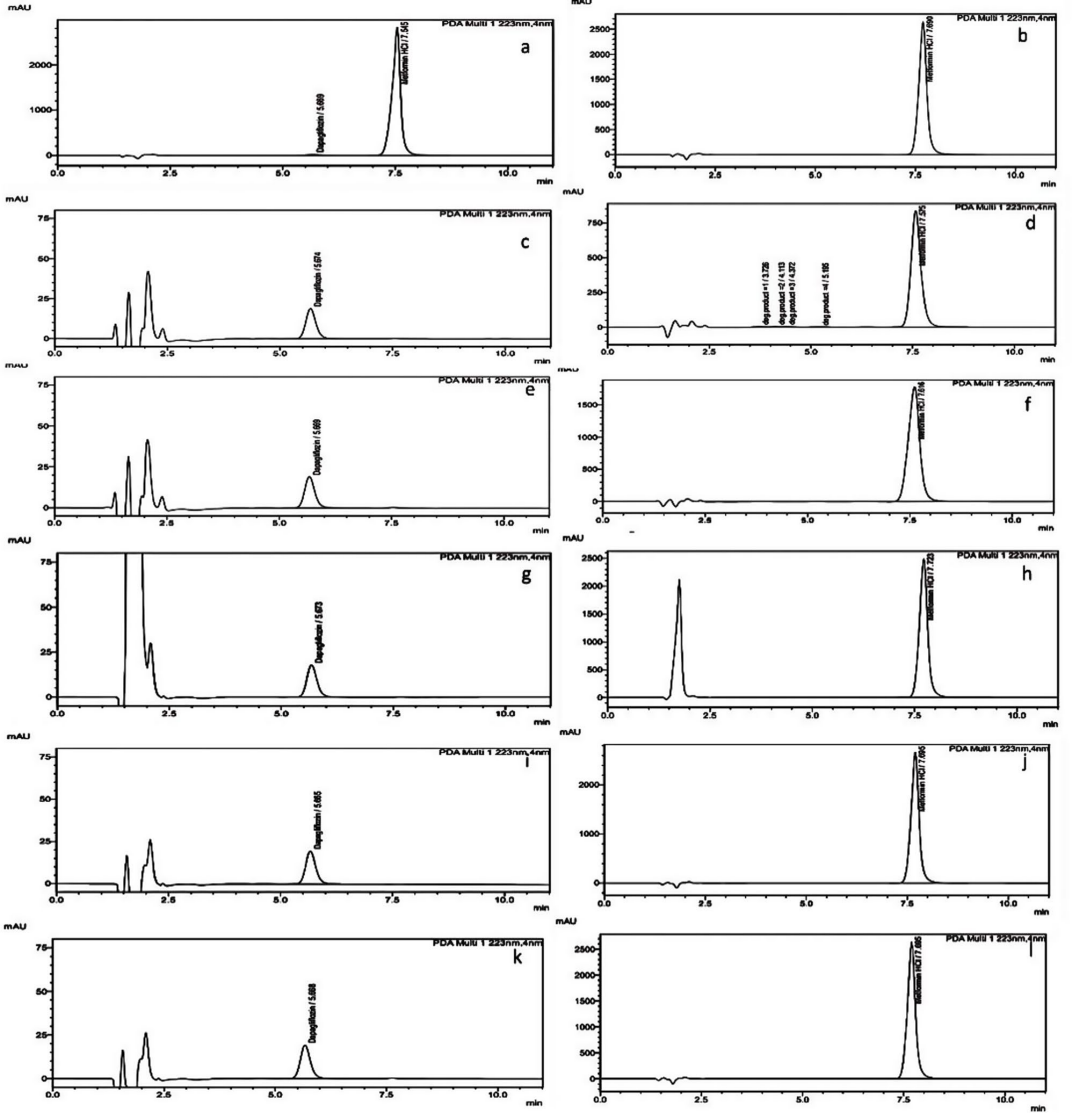
Chromatograms of **a**: unstressed solution 5 µg/mL DAP, **b**: unstressed solution 500 µg/mL MET, **c**: DAP alkaline hydrolysis, **d**: MET alkaline hydrolysis, **e**: DAP acidic hydrolysis, **f**: MET acidic hydrolysis, **g**: DAP oxidative degradation, **h**: MET oxidative degradation, **i**: DAP photo degradation, **j**: MET photo degradation, **k**: DAP heat degradation and **l**: MET heat degradation. All under the specified chromatographic conditions

The specificity of the developed approach was confirmed by comparing the chromatograms of unstressed of 5 µg/mL DAP and 500 µg/mL MET solutions (Fig. 2, a &b) with solutions which were exposed to five degradation conditions separately.

Stability studies carried out using five forced degradation conditions (acidic, alkaline, photo, heat, and oxidation degradations) as per ICH guidelines [47].

#### Alkaline hydrolysis (0.1 N sodium hydroxide for 5 h at 80 °C)

DAP chromatogram shows no alkaline degradation (Fig. 2, c), while MET chromatogram shows significant alkaline degradation (Fig. 2, d) and appearance of four small peaks after exposed to 0.1 N Sodium hydroxide for 5 h at 80 °C.

The chromatogram of MET alkaline degradation (Fig. 2, c) showed very high sensitivity, as it gave the maximum %degradation about 61.107% and presence of four degradation peaks as presented in Table 2.

#### Acid hydrolysis (0.1 N hydrochloric acid for 5 h at 80 °C)

The chromatogram of DAP shows no degradation in acidic media (Fig. 2, e). While, MET showed a very slight acidic degradation as the MET %recovery was reduced about 5.803% as presented in Table 2 and (Fig. 2, f).

#### Oxidative hydrolysis (3% hydrogen peroxide for 5 h)

DAP was giving 7.86% degradation using 3% H_2_O_2_ for 5 h as shown in Table 2 and (Fig. 2, g). While MET chromatogram shows slight degradation as the %degradation was 4.811% as result of oxidative degradation conditions as presented in Table 2 and (Fig. 2, h).

#### Photo degradation (day light for 6 h)

DAP and MET chromatograms showed no degradation after exposed to photo degradation conditions as the peak area doesn’t reduce for both drugs and no new degradation peaks appeared as shown in Table 2 and (Fig. 2, i & j).

#### Heat hydrolysis (80 °C for 6 h.)

DAP and MET chromatograms show no heat degradation as presented in Table 2 and (Fig. 2, k &l).

**Table 2:** Forced degradation results of DAP and MET

Conditions	Drug	Area	% Recovery	% Degradation	New peaks
Acid	DAP	301,964	97.925	2.075	--
	MET	35,193,596	94.197	5.803	--
Alkaline	DAP	296,901	96.283	3.717	--
	MET	14,531,180	38.893	61.107	4 new peaks
Heat	DAP	301,369	97.732	2.268	--
	MET	37,007,941	99.053	0.947	--
Photodegradation	DAP	308,138	99.927	0.073	--
	MET	37,300,529	99.836	0.164	--
Oxidation	DAP	284,114	92.136	7.864	--
	MET	35,564,242	95.189	4.811	--

### Proposed degradation pathways based on literature

Although experimental determination of degradation pathways was not performed, we propose the likely degradation pathways based on literature reviews and known chemical properties of the compounds.

Based on published literature, DAP is susceptible to hydrolytic degradation, particularly under acidic or basic conditions, which can lead to the cleavage of the O-glucoside bond. Hydrolysis results in the formation of aglycone and glucose [48, 49].

MET is known to undergo degradation via oxidative pathways, especially under stress conditions. The biguanide structure is prone to oxidation, leading to the formation of cyanoguanidine derivatives. Hydrolytic degradation is also possible, albeit less common, yielding derivatives such as 1-methylbiguanide [50].

### Assay of Dexigloflozin plus ^®^ tablets

**Fig. 3:**
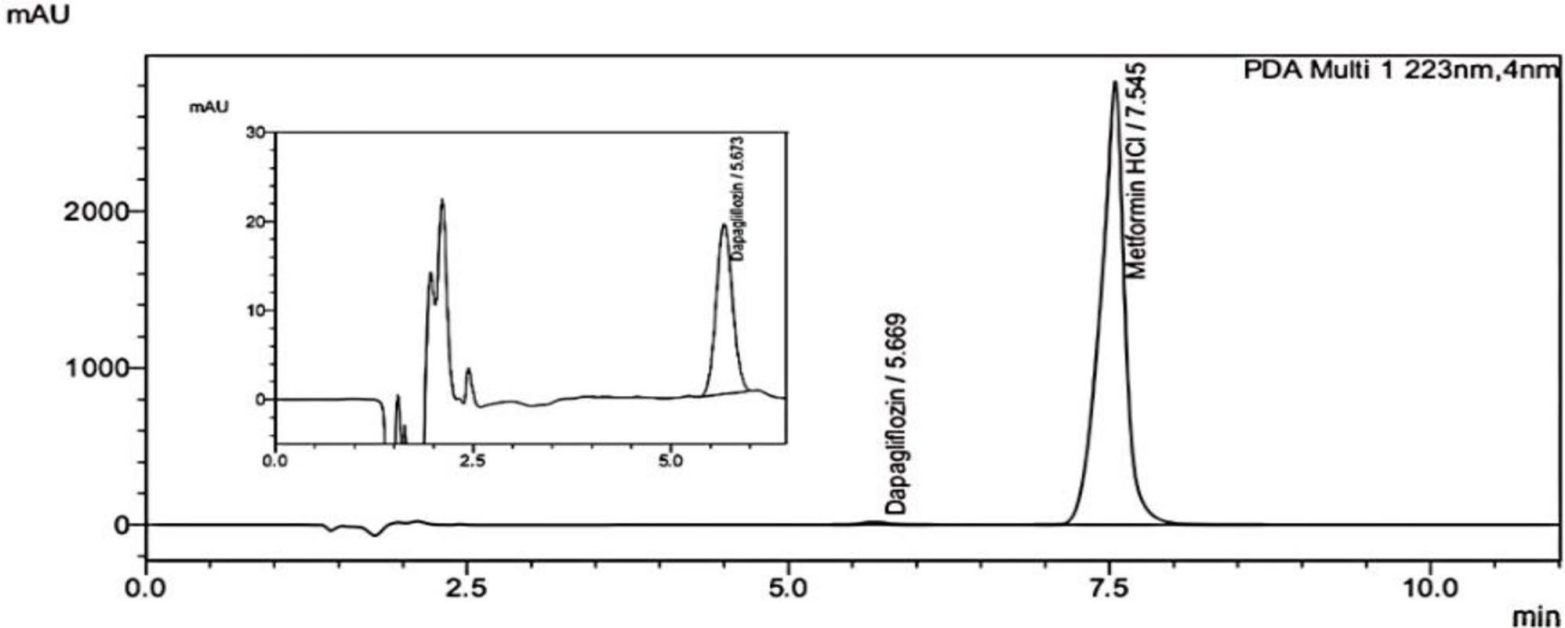
Separated chromatogram of Dexigloflozin plus^®^ containing 5 µg/mL DAP &500 µg/mL MET under the specified chromatographic conditions. The inset show the peak of DAP

The developed approach was efficiently applied for the separation and estimation of DAP and MET in Dexigloflozin plus^®^ tablets which is presents in Egyptian markets as shown in Fig. 3; Table 3 showed the assay results for determination of DAP and MET and good agreement with labeled claim.

The assay results of developed and reported [34] approaches were compared using T-test and F-test for accuracy and precision at 95% confidence level, respectively as shown in Table 3. The calculated values did not exceed the tabulated values, confirms that there was no significant difference between the developed and published [34] methods.

**Table 3:** Assay results of Dexigloflozin plus^®^ tablets using the developed and published [34] methods

	Proposed method		Reported method [34]	
	DAP	MET	DAP	MET
Mean (Ẋ)	101.057	101.320	100.734	100.965
S. D	0.355	0.459	0.371	0.521
%RSD	0.351	0.453	0.368	0.516
T_cal_	1.543	1.252	T _tab_	2.228
F_cal_	1.090	1.287	F _tab_	5.050

S.D: standard deviation, RSD: relative standard deviation, t_cal_: calculated t-value,

t_tab_: tabulated t-value, F_cal_: calculated F-value, F_tab_: tabulated F-value

### Results of greenness assessment

The Eco-Scale, Complex Modified GAPI and Analytical GREEnness (AGREE) methodologies were used to evaluate the suggested MLC method’s greenness [51–53]. While Complex MoGAPI [43] gives a pictogram-based evaluation of greenness across several analytical stages, with an emphasis on reagent toxicity and energy requirements, the eco-scale assesses the environmental impact based on chemical usage, energy consumption, and waste creation [42]. The AGREE approach provides a thorough framework for assessing sustainability from a variety of angles, including as economic and safety considerations [44]. The 12 principles of green analytical chemistry were assessed using the AGREE software, generating a circular pictogram that highlights areas of greenness and areas for potential improvement [44].

Greenness assessment using Complex GAPI not provide a total score to determine whether the method is truly green. So, a modified version, Complex MoGAPI, addresses this limitation was used for greenness assessment of the proposed and published methods. Complex MoGAPI and Analytical GREEnness (AGREE) methodologies were used also to compare the greenness of proposed method with that of previously reported methods [33, 35].

**Fig. 4:**
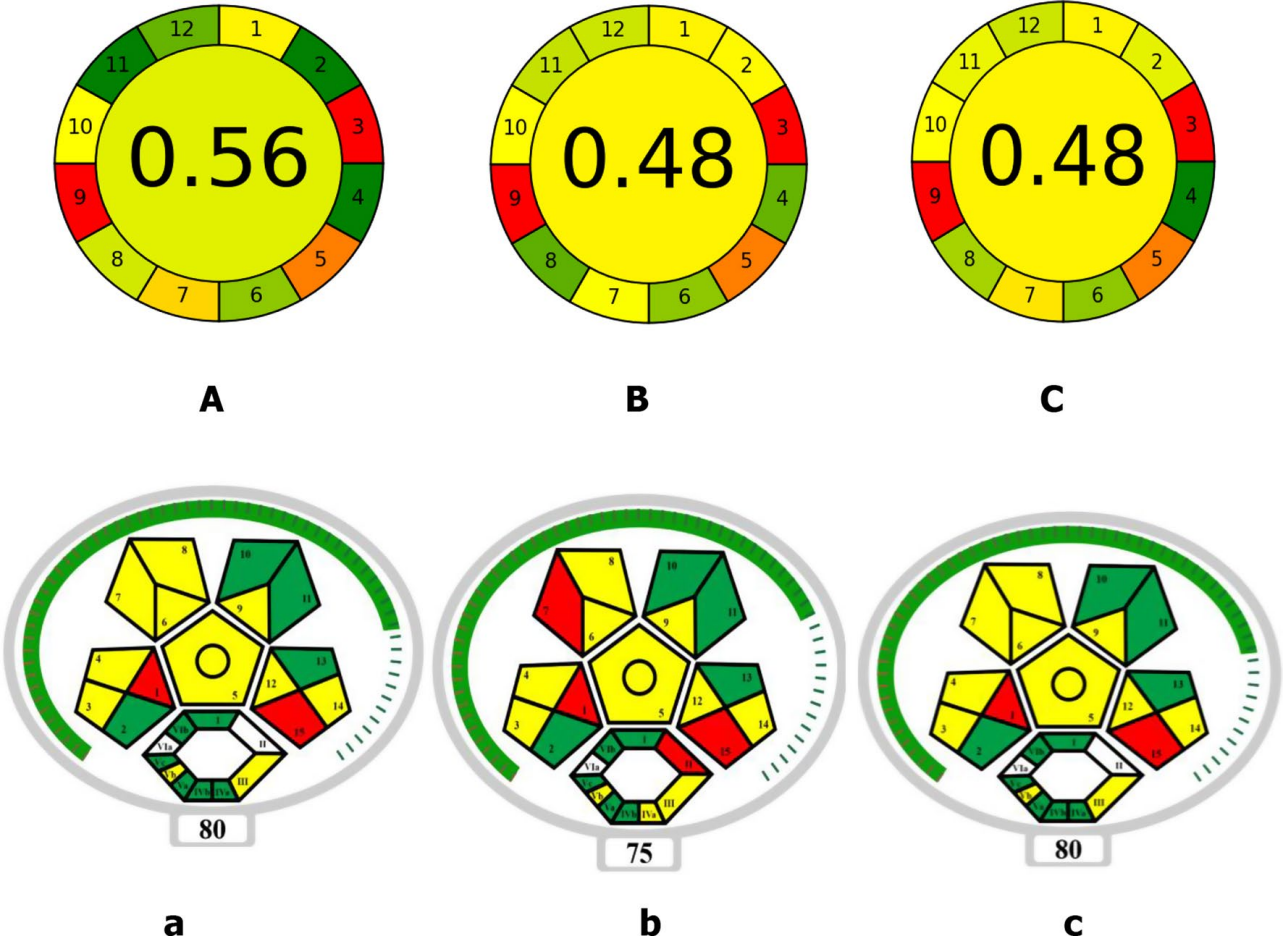
Results for greenness assessment using AGREE method (**A**-**C**); where **A**: for the proposed method, **B** and **C** for published methods respectively [33 & 35] and using Complex MoGAPI method (**a**-**c**); where a for the proposed method, **b** and **c** for published methods respectively [33, 35]

Figure 4 shows the results from the two evaluation tools for the proposed method and two reported methods [33, 35]. As shown in Fig. 4**(A-C)** the score of AGREE method for the proposed method was higher than that of the two previously published methods [33, 35]. Also, Fig. 4**(a-c)** shows that the total score of Complex MoGAPI for the proposed method was higher than that of published method [33] according to comparative analysis.

In addition to demonstrating the environmental friendliness of our methodology, this thorough assessment supports sustainable analytical chemistry practices and the development of more environmentally friendly pharmaceutical analysis techniques.

Analytical eco-scale approach was carried out for greenness evaluation [42], the sum of total penalty points was determined using Eq. (3) for the all procedure and the results shows that the developed approach was excellent green with analytical eco-scale 79 as shown in (Table 4).

3 $$\begin{aligned}{\text{Analytical}}\;{\text{Eco}} - {\text{Scale}} = 100 \\  - {\text{Sum }}\;{\text{of}}\;{\text{total}}\;{\text{penalty}}\;{\text{points}}\end{aligned}$$

**Table 4:** Results for calculation of analytical eco-scale total score [42]

Reagents	Amount	Signal word	No of pictograms	Sub-total PP
SLS	< 10 ml = 1	0	0	0
Phosphoric acid	< 10 ml = 1	Danger = 2	1	2
TEA	< 10 ml = 1	Danger = 2	3	6
2-Propanol	< 10 ml = 2	Danger = 2	2	4
**Total PP for reagents**				**12**
**Instrument**	HPLC = 2			2
Waste	1-10 ml = 2			2
Treatment of waste	No treatment(waste) = 3			3
**Total PP for HPLC instrument**				**7**
**Centrifugation = 2**				2
**Total PP for other instruments**				**2**
Total PP				**21**
Analytical Eco-Scale total score:				**79(**excellent green)

> 75 represents excellent green analysis,

> 50 represents acceptable green analysis,

< 50 represents inadequate green analysis

The penalty points of the analytical eco-scale were calculated based on reagent quantities, hazard statements for each chemical, energy consumption (heating during sample preparation), and waste generation [42]. The final score was calculated and compared to established benchmarks for green analytical methods [42].

**Fig. 5:**
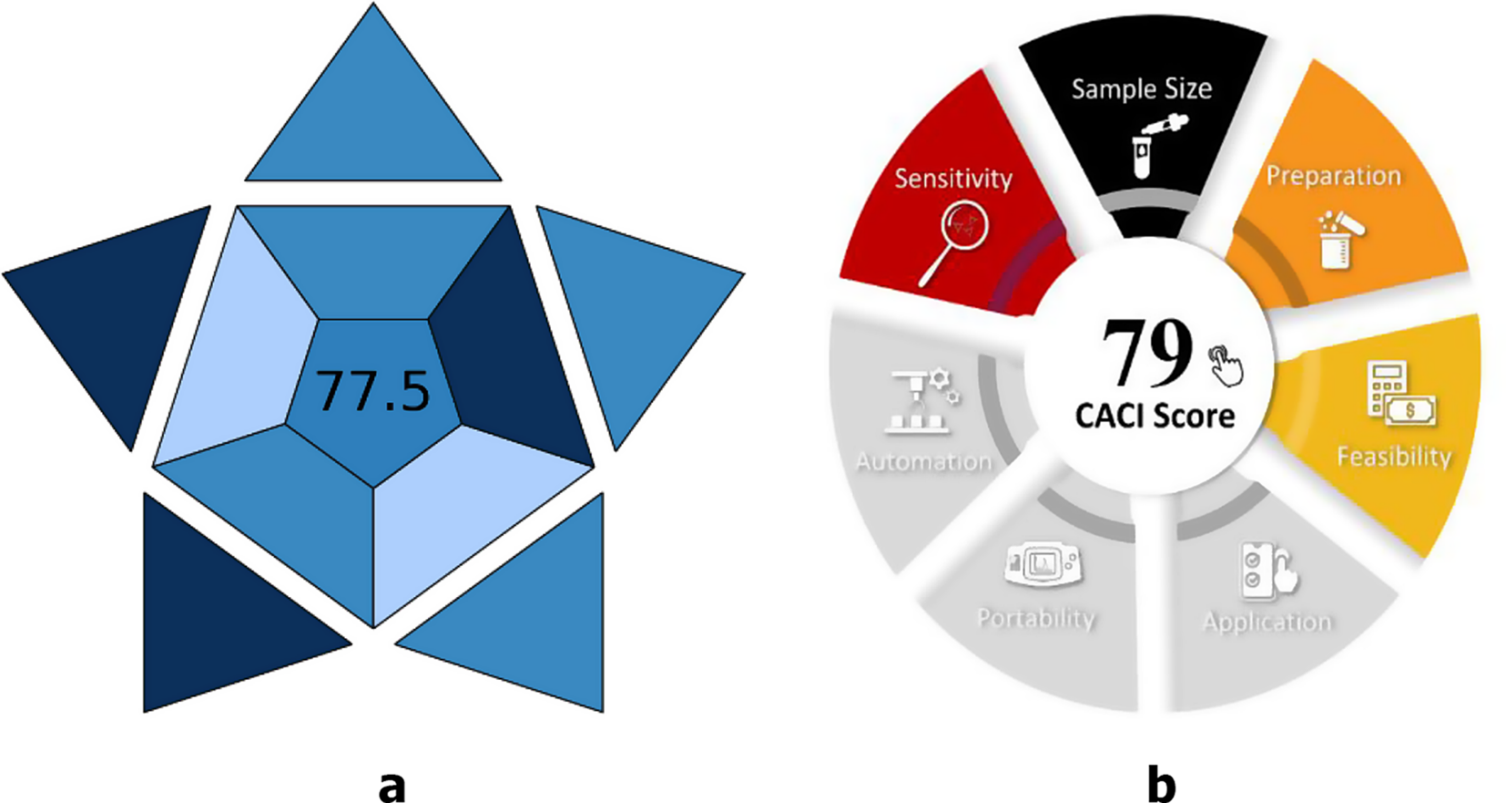
The practicality assessment of the developed method using **a**: BAGI and **b**: CACI

Green methods are not necessarily practical, so the practicality of the developed method was evaluated using BAGI [54] and CACI [55] methods as shown in Fig. 5**(a & b)**. The proposed method had low complexity and cost according to CACI [55] and had high greenness and bench suitability according to BAGI [54].

Table S4 presents a comparison between the proposed (MLC) and the two stability indicating methods [33, 35]. The comparison confirms the superiority and advantages of proposed (MLC) method.

The reduced solvent consumption and waste generation contribute to lower operational costs and reduced environmental impact. The method’s robustness, demonstrated through validation parameters (accuracy, precision, linearity), makes it suitable for routine analysis of fixed-dose combination tablets.

A comparison between the advantages and disadvantages of the present MLC technique and another technique was shown in (Table S5) [56–58].

While advanced techniques offer certain advantages, our method provides a valuable alternative due to its key advantages, e.g., simplicity, cost-effectiveness, accessibility in resource-limited settings.

## Conclusion

A sensitive and fast micellar liquid chromatographic (MLC) approach for green separation of DAP and MET in pure and in tablet forms. The developed MLC was used a hybrid micellar mobile phase composed of a small amount of organic modifier with surfactant above the (CMC) to increase the elution strength and efficiency. Stability studies of DAP and MET were performed to approve the specificity of the developed approach after exposure to five degradation conditions. For the greenness assessment the analytical eco-scale, ComplexMoGAPI and AGREE methods were applied.

## Electronic supplementary material

Below is the link to the electronic supplementary material.

Supplementary Material 1

## Data Availability

Data is provided within the manuscript or supplementary information files.
