# Supplementary material for: Stability indicating green micellar liquid chromatographic method for simultaneous analysis of Metformin and dapagliflozin in their tablets
Source: BMC Chem. 2025 Jun 21;19(1):175. doi: 10.1186/s13065-025-01537-8 (PMC12181907; doi:10.1186/s13065-025-01537-8)
Supplement: Supplementary file 1 — Supplementary Material 1 [file 13065_2025_1537_MOESM1_ESM.docx]

**Supplementary Figures**

**Figure S1:** Chemical structures of Dapagliflozin (a) and Metformin (b)

**Figure S2**: Chromatograms of a: Dexigloflozin plus^®^ 5 /500mg tablet, b: standard solution of (5µg/mL DAP & 500µg/mL MET) in pure forms, c: placebo of Dexigloflozin plus^®^ tablet, d: diluent (solvent). All under the specified chromatographic conditions.


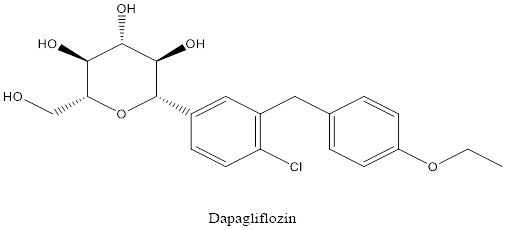

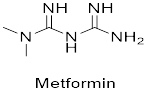


b

a

**Figure S1:** Chemical structures of Dapagliflozin (a) and Metformin (b)


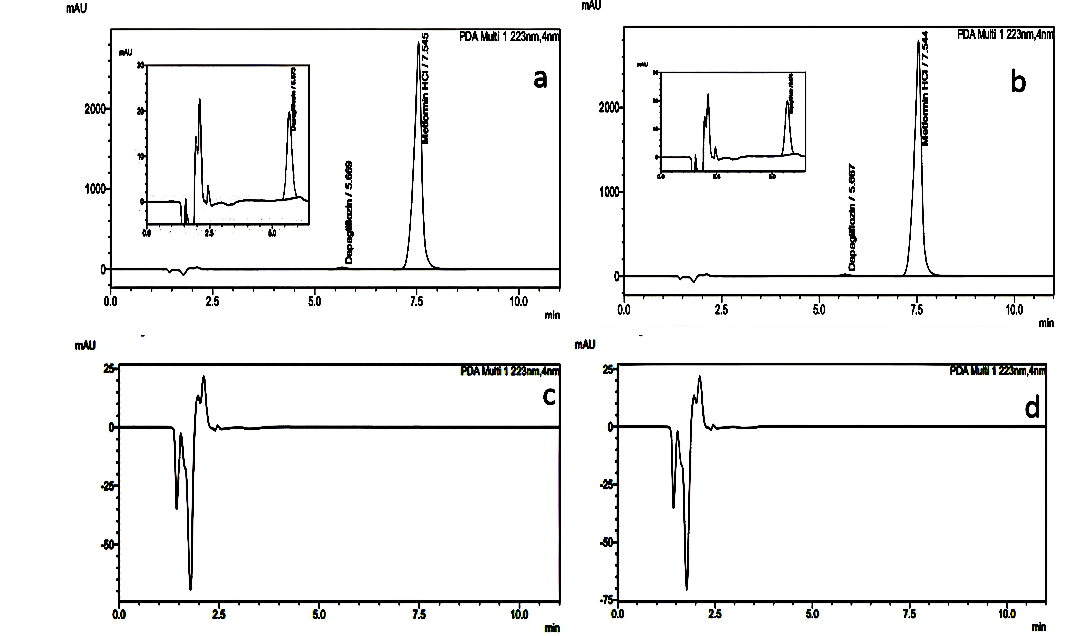


**Figure S2**: Chromatograms of a: Dexigloflozin plus^®^ 5 /500mg tablet, b: standard solution of (5µg/mL DAP & 500µg/mL MET) in pure forms, c: placebo of Dexigloflozin plus^®^ tablet, d: diluent (solvent). All under the specified chromatographic conditions.

**Supplementary Tables**

**1- Table S1:** System suitability parameters for DAP and MET determination of at the optimal separation conditions.

**2- Table S2:** Accuracy and precision results for estimation of DAP and MET.

3- **Table S3:** Robustness results for estimation of DAP and MET.

4- **Table S4**: Comparison between the proposed stability indicating (MLC) and the two reported stability indicating methods.

**5- Table S5:** Comparison between the advantages and disadvantages of the present MLC technique and another three techniques.

**Table S1:** System suitability parameters for DAP and MET determination of at the optimal separation conditions.

| Parameter | DAP | MET |
| --- | --- | --- |
| Retention time (min.) | 5.678 | 7.599 |
| Number of theoretical plates | 3375 | 7986 |
| Tailing factor t_f_ | 1.076 | 0.848 |
| Retention factor (k) | 3.224 | 4.296 |
| Resolution | 5.254 | |
| Selectivity factor | 1.332 | |

| Taken Conc. | | Accuracy | | | | | | | Intra-day precision | | | | | | | | | | | | Inter-day precision | | | | | | | | | | | |
| --- | --- | --- | --- | --- | --- | --- | --- | --- | --- | --- | --- | --- | --- | --- | --- | --- | --- | --- | --- | --- | --- | --- | --- | --- | --- | --- | --- | --- | --- | --- | --- | --- |
| (µg/ mL) | | DAP | | | | MET | | | DAP | | | | | | MET | | | | | | DAP | | | | | | MET | | | | | |
| DAP | MET | Obtained Conc. | | Recovery % | | Obtained Conc. | | Recovery % | Mean conc. found | | S.D. | | %RSD | | Mean conc. found | | S.D. | | %RSD | | Mean conc. found | | S.D. | | %RSD | | Mean conc. found | | S.D. | | | %RSD |
|  |  | (µg/ mL) | |  |  | (µg/ mL) | |  |  |  |  |  |  |  |  |  |  |  |  |  |  |  |  |  |  |  |  |  |  |  |  |  |
| 2.5 | 250 | 2.496 | | 99.837 | | 251.4104 | | 100.564 | 2.498 | | 0.003 | | 0.121 | | 250.868 | | 0.767 | | 0.306 | | 2.498 | | 0.004 | | 0.175 | | 250.041 | | 0.101 | | | 0.041 |
| 5 | 500 | 5.022 | | 100.44 | | 500.804 | | 100.161 | 5.036 | | 0.019 | | 0.379 | | 499.875 | | 1.313 | | 0.263 | | 5.031 | | 0.05 | | 0.996 | | 500.239 | | 0.161 | | | 0.032 |
| 6 | 600 | 5.962 | | 99.367 | | 596.27 | | 99.378 | 5.97 | | 0.011 | | 0.184 | | 597.281 | | 1.429 | | 0.239 | | 6.008 | | 0.021 | | 0.353 | | 600.573 | | 0.509 | | | 0.085 |
| Mean % | |  |  | | 99.881 | |  |  | 100.034 |  | |  | |  | |  | |  | |  | |  | |  | |  | |  | |  |  | |
| S.D. | |  |  | | 0.538 | |  |  | 0.603 |  | |  | |  | |  | |  | |  | |  | |  | |  | |  | |  |  | |
| RSD % | |  |  | | 0.539 | |  |  | 0.603 |  | |  | |  | |  | |  | |  | |  | |  | |  | |  | |  |  | |

**Table S2:** Results of accuracy and precision evaluation for DAP and MET determination

S.D: standard deviation, % RSD: percent relative standard deviation

**Table S3:** Robustness results for estimation of DAP and MET

| Parameters | Conditions | Mean % recovery | | S.D. | | %RSD | |
| --- | --- | --- | --- | --- | --- | --- | --- |
|  |  | DAP | MET | DAP | MET | DAP | MET |
| pH | 3.2 | 101.235 | 100.985 | 0.525 | 0.236 | 0.519 | 0.234 |
|  | *3.3 |  |  |  |  |  |  |
|  | 3.4 |  |  |  |  |  |  |
| Flow rate | 0.9 mL/min | 99.894 | 100.368 | 0.726 | 0.302 | 0.727 | 0.301 |
|  | *1 mL/min |  |  |  |  |  |  |
|  | 1 mL/min |  |  |  |  |  |  |
| Temp | 39 °C | 100.238 | 101.355 | 0.445 | 0.568 | 0.444 | 0.560 |
|  | *40 °C |  |  |  |  |  |  |
|  | 41 °C |  |  |  |  |  |  |

*Indicates the optimum separation conditions

**Table S4**: Comparison between the proposed stability indicating (MLC) and the two reported stability indicating methods[33, 35].

|  | Proposed Method | Reported method [33] | Reported method [35] |
| --- | --- | --- | --- |
| Type of analysis | MLC | RP-HPLC | RP-HPLC |
| Stability indicating | Yes | Yes | Yes |
| Linearity range | DAP: (0.2-7µg/mL)  MET (50-700µg/mL) | DAP: (1.25-7.5μg/mL) MET: (125-750μg/mL) | DAP: (2–10μg/mL)  MET: (100–500μg/mL) |
| LOD & LOQ for DAP | LOD of DAP 0.085 and LOQ DAP 0.255 | Not mentioned | LOD of DAP: 0.06μg/mL LOQ for DAP:0.185μg/mL |
| Mobile phase | 50 gm of SLS in 500mL purified water with 100mL of 2-Propanol and 3mL triethylamine, completed to 1000mL purified water. | 60% phosphate buffer (pH: 3) and 40% acetonitrile. | Methanol: water  (75:25) % v/v pH:3 |

**Table S5:** Comparison between the advantages and disadvantages of the present MLC technique and another three techniques.

| **Technique** | **Advantages** | **Disadvantages** |
| --- | --- | --- |
| **Proposed MLC** | - Environmentally friendly (uses aqueous micellar mobile phases, reducing organic solvents)  - Good separation of polar and non-polar compounds  - Can eliminate sample pretreatment steps  - Compatible with UV detection | -Requires careful optimization of micelle concentration.  -Not compatible with all column types. |
| **Conventional HPLC**  **[56]** | - Highly reproducible and robust  -Wide applicability in pharmaceutical analysis  - Compatible with many detectors (UV, MS, fluorescence) | - Uses large volumes of organic solvents (environmental and cost concerns).  - May require complex sample preparation  - Higher operational cost |
| **Spectrophotometry**  **[57]** | - Simple and inexpensive- Good for routine analysis  -Rapid detection of reaction endpoints | -Lower selectivity compared to chromatographic methods  - Prone to interferences from other absorbing species |
| **Colorimetry**  **[58]** | - Easy to use and low-cost equipment  - Visual or instrumental detection possible | - Less sensitive and precise than spectrophotometry or HPLC  - Subjective interpretation if visual  - Narrow concentration range of accuracy |
